# Supplementary material for: Should Age-Dependent Absolute Risk Thresholds Be Used for Risk Stratification in Risk-Stratified Breast Cancer Screening?
Source: J Pers Med. 2021 Sep 15;11(9):916. doi: 10.3390/jpm11090916 (PMC8469877; doi:10.3390/jpm11090916)
Supplement: Supplementary file 1 [file jpm-11-00916-s001.zip › jpm-1349724-supplementary.pdf]

Supplementary Materials

Table S1. The population average absolute risk of developing breast cancer over the remaining lifetime to age 80 years and over five and ten years in women in Canada. (This is based on breast cancer registration, breast cancer deaths and all other cause of deaths, Canada Statistics, 2012-2016).

Current age	Remaining life time risk (from current age to age 80)	10-year absolute risk	5-year absolute risk
30	0.1009	0.0041	0.0013
31	0.1007	0.0048	0.0015
32	0.1006	0.0055	0.0018
33	0.1004	0.0063	0.0021
34	0.1002	0.0072	0.0024
35	0.0999	0.0081	0.0028
36	0.0996	0.0091	0.0032
37	0.0992	0.0101	0.0037
38	0.0987	0.0112	0.0042
39	0.0982	0.0123	0.0048
40	0.0976	0.0133	0.0053
41	0.0969	0.0143	0.0059
42	0.0961	0.0153	0.0064
43	0.0952	0.0163	0.0070
44	0.0942	0.0173	0.0075
45	0.0931	0.0181	0.0081
46	0.0918	0.0189	0.0086
47	0.0905	0.0196	0.0090
48	0.0891	0.0203	0.0094
49	0.0876	0.0210	0.0098
50	0.0860	0.0217	0.0102
51	0.0844	0.0225	0.0105
52	0.0827	0.0233	0.0108
53	0.0809	0.0242	0.0110
54	0.0790	0.0252	0.0113
55	0.0772	0.0263	0.0118
56	0.0753	0.0274	0.0122
57	0.0733	0.0287	0.0128
58	0.0713	0.0301	0.0135
59	0.0692	0.0314	0.0142
60	0.067	0.0327	0.0149
61	0.0647	0.0339	0.0156
62	0.0622	0.0351	0.0163
63	0.0596	0.0363	0.0171
64	0.0569	0.0371	0.0178
65	0.0540	0.0378	0.0185
66	0.0510	0.0381	0.0191
67	0.0478	0.0383	0.0196
68	0.0444	0.0381	0.0200
69	0.0410	0.0378	0.0203

70	0.0375	0.0375	0.0203
71	0.0338	0.0370	0.0202
72	0.0300	0.0363	0.0199
73	0.0262	0.0356	0.0194
74	0.0223	0.0349	0.0189
75	0.0186	0.0342	0.0186
76	0.0148	0.0336	0.0183
77	0.0112	0.033	0.0180
78	0.0075	0.0324	0.0179
79	0.0038	0.0316	0.0177

Figure S1. 5-year absolute risk for breast cancer in women in Canada (2012-2016), where risk thresholds for the 'high risk' and 'higher than average' risk categories are set to be equivalent to remaining lifetime risk of 25% and 15% if determined at age 40.

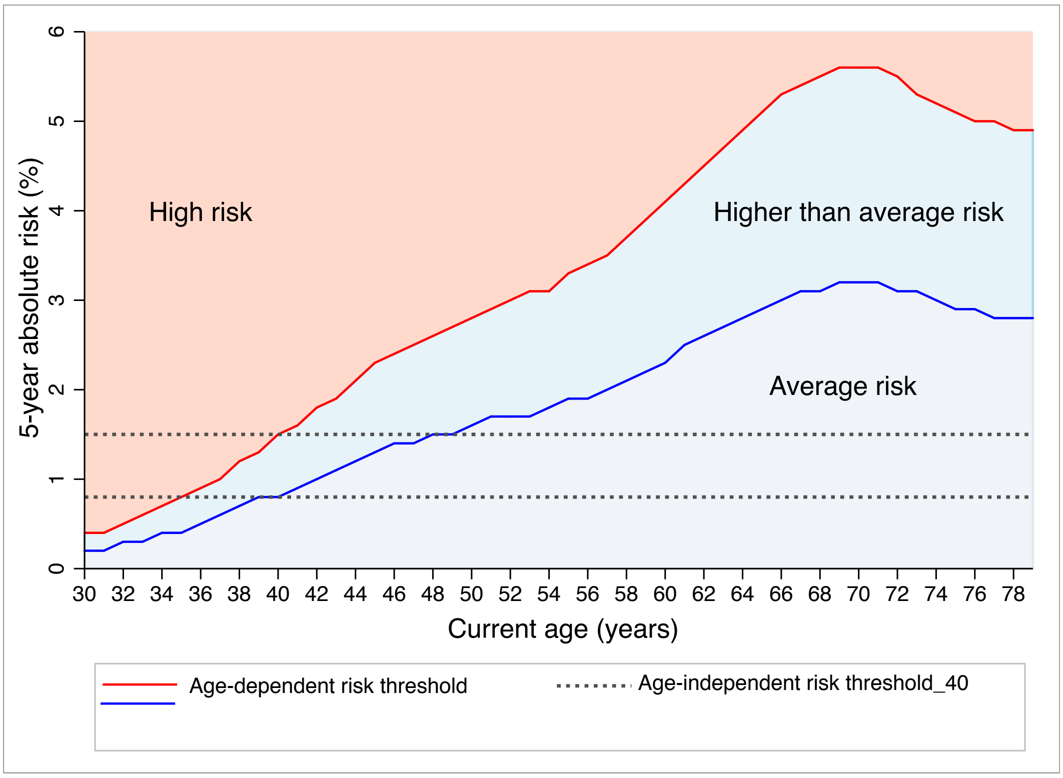

Grey dotted lines: 10-year absolute risks of 1.5% and 0.8% when assessed at age 40 are equivalent to 25% and 15% remaining lifetime risks from age 40 to 80.
